# Post-Operative Narrow Complex Tachycardia: What is the Mechanism?

**Published:** 2010-07-20

**Authors:** Arash Arshi, Samir Saba

**Affiliations:** Cardiovascular Institute, University of Pittsburgh Medical Center, Pittsburgh, PA

**Keywords:** Junctional tachycardia, narrow complex tachycardia, valvular surgery

## Case presentation

**Figure 1:**
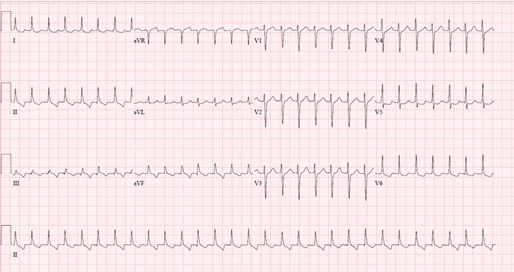
A 12-lead surface electrocardiogram of the narrow-complex tachycardia with heart rate 173. Retrograde p-waves are seen within the T-waves in a 1:2 ratio with the QRS complexes and are best seen in the inferior leads.

A 58 year old woman with symptomatic severe bicuspid aortic valve stenosis underwent aortic valve replacement with a mechanical prosthesis. On post-operative day four, the patient developed sudden onset of sustained narrow complex tachycardia with a heart rate of 170 - 180 (Figure 1). She complained of palpitations, but her blood pressure remained stable. The tachycardia was able to be briefly terminated with direct current cardioversion, revealing underlying sinus rhythm, but would recur within seconds. The tachyarrhythmia was resistant to treatment with beta blockers, calcium channel blockers, amiodarone, and digoxin and remained incessant for 48 hours. What is the rhythm, and how should the patient be managed?

## Commentary

**Figure 2:**
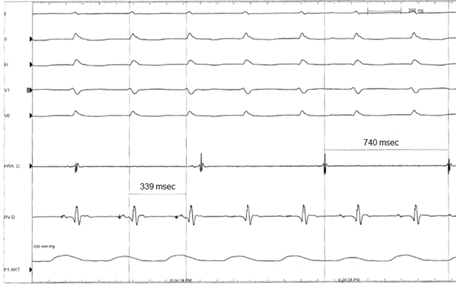
Intracardiac recording of the arrhythmia at presentation to the electrophysiology laboratory. The tracings from top to bottom represent surface leads I, II, III, V1, and V6 as well as intracardiac recordings from the high right atrial and the His catheters. An arterial catheter tracing is also shown at the bottom of the figure. Note the dissociation between the atrial signal and the His/ventricular signals.

**Figure 3:**
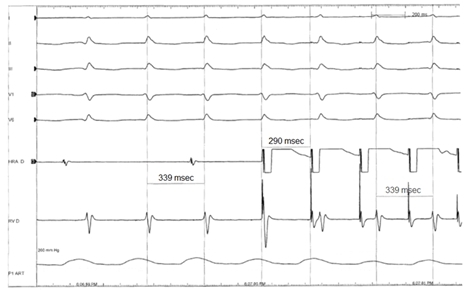
Surface and intracardiac recordings are similar to figure 2. Atrial pacing from high right atrium demonstrates atrial dissociation from the junctional rhythm confirming the lack of participation of atrial tissue in the rhythm mechanism.

Initial evaluation of the presenting arrhythmia (Figure 1) revealed a narrow complex tachycardia at a rate of 173 beats per minute, consistent with supraventricular tachycardia. The unusual feature of the arrhythmia was its incessant nature, whereby it would spontaneously reinitiate within seconds after successful cardioversion. A careful examination of the surface electrocardiogram in Figure 1 reveals the absence of 1:1 ventriculo-atrial (V-A) association, whereby one atrial signal could be seen for every two QRS complexes. An electrophysiology study was performed to ascertain the mechanism of the tachycardia. Quadripolar catheters were placed in the high right atrium, His position, and right ventricular apex. Figure 2 shows the presenting intracardiac recordings. The AA cycle length was 740 msec and the VV cycle length was 339 msec, with a His recording preceding each QRS complex (HV interval = 44 msec). Ventricular pacing consistently terminated the tachycardia with rapid re-initiation within seconds. Atrial pacing did not affect the ventricular rhythm (Figure 3). The finding of V-A dissociation combined with regular narrow complex ventricular beats preceded each by a His recording was consistent with an accelerated junctional tachycardia. Atrial pacing from the high right atrium was dissociated from the ventricular rhythm, confirming that the tachycardia did not involve atrial tissue. Ablation was not performed given the high risk of inducing permanent complete heart block with ablation of junctional arrhythmias. The patient was continued on amiodarone and over the next three days the junctional tachycardia intermittently terminated for increasing periods of time, and the patient eventually maintained normal sinus rhythm. She was discharged on amiodarone, and has had no recurrences of the arrhythmia in over two months of follow up.

Accelerated junctional tachycardia in the post-cardiac surgery period has been described in the pediatric and adult congenital populations, and has been associated with significant morbidity [1]. However, in the general adult population, accelerated junctional tachycardia is exceedingly rare with few case reports [2,3], and to our knowledge, has not been reported in the post-operative setting. This is in distinction to nonparoxysmal junctional tachycardia, which is associated with myocardial ischemia, digitalis toxicity, or after cardiac surgery, and is associated with a slower rate between 70 and 120 bpm [4]. In the pediatric population, first-line treatment for post-operative junctional tachycardia includes hypothermia and antiarrhythmic drug therapy with amiodarone [5]. A recent observational report has suggested that the use of dexmedetomidine may have a potential therapeutic role in the acute phase of perioperative atrial and junctional tachyarrhythmias [6]. Radiofrequency ablation is not recommended except in refractory cases, as it is associated with an unacceptably high risk of complete heart block. Whether cryoablation offers an acceptable alternative is possible but unproven. In general, post-operative junctional tachycardia is self-limited and recurrences are uncommon.

In conclusion, we report the case of a patient who developed incessant, accelerated junctional tachycardia few days after aortic valve surgery. This condition is self-limited and can be treated acutely with anti-arrhythmic medications such as amiodarone. Recognizing this condition and treating it conservatively are important to ensure a favorable clinical outcome.
